# The Sum of Its Parts—Effects of Gastric Distention, Nutrient Content and Sensory Stimulation on Brain Activation

**DOI:** 10.1371/journal.pone.0090872

**Published:** 2014-03-10

**Authors:** Maartje S. Spetter, Cees de Graaf, Monica Mars, Max A. Viergever, Paul A. M. Smeets

**Affiliations:** 1 Image Sciences Institute, University Medical Center Utrecht, Utrecht, The Netherlands; 2 Division of Human Nutrition, Wageningen University, Wageningen, The Netherlands; INRA, France

## Abstract

During food consumption the brain integrates multiple interrelated neural and hormonal signals involved in the regulation of food intake. Factors influencing the decision to stop eating include the foods' sensory properties, macronutrient content, and volume, which in turn affect gastric distention and appetite hormone responses. So far, the contributions of gastric distention and oral stimulation by food on brain activation have not been studied. The primary objective of this study was to assess the effect of gastric distention with an intra-gastric load and the additional effect of oral stimulation on brain activity after food administration. Our secondary objective was to study the correlations between hormone responses and appetite-related ratings and brain activation. Fourteen men completed three functional magnetic resonance imaging sessions during which they either received a naso-gastric infusion of water (stomach distention), naso-gastric infusion of chocolate milk (stomach distention + nutrients), or ingested chocolate-milk (stomach distention + nutrients + oral exposure). Appetite ratings and blood parameters were measured at several time points. During gastric infusion, brain activation was observed in the midbrain, amygdala, hypothalamus, and hippocampus for both chocolate milk and water, i.e., irrespective of nutrient content. The thalamus, amygdala, putamen and precuneus were activated more after ingestion than after gastric infusion of chocolate milk, whereas infusion evoked greater activation in the hippocampus and anterior cingulate. Moreover, areas involved in gustation and reward were activated more after oral stimulation. Only insulin responses following naso-gastric infusion of chocolate milk correlated with brain activation, namely in the putamen and insula. In conclusion, we show that normal (oral) food ingestion evokes greater activation than gastric infusion in stomach distention and food intake-related brain areas. This provides neural evidence for the importance of sensory stimulation in the process of satiation.

**Trial Registration:**

ClinicalTrials.gov NCT01644539.

## Introduction

Obesity prevalence has increased dramatically the last decades [1], as a result of overconsumption [2]. Key elements in the control of food intake are satiation and satiety. Satiation refers to the process which leads to meal termination [3]. It is a complex process which is determined by many different factors, including the foods' sensory properties, macronutrient content, and volume, which influence hormone levels and gastric distention [4]. Satiety is the ensuing state of satisfaction after the meal and is related to the post-ingestive consequences of consumption, such as digestion and hormone signaling. Gastric [5], [6] as well as oral [7]–[10] stimulation contribute separately and in conjunction with meal termination [11]. For example, higher viscosity leads to decreased intake [12], and increased oro-sensory exposure can lower the intake of sweet drinks [13].

However, gastric processes have been proposed to be equally important for meal termination. This includes stomach distention by meal volume and weight, related hormone responses, and macro-nutrient induced duodenal hormone release, which slows gastric emptying [14], [15].

Food consumption involves several brain areas, including those subserving sensory perception, in particular vision, taste, oral sensations and smell processing. Taste information travels from the tongue to the brainstem nucleus of the solitary tract, via the thalamus to the primary taste cortex in the frontal operculum and the mid- and anterior insula. From here, taste neurons project to the ventral insula and the medial and lateral orbitofrontal cortex [16]–[18]. Olfactory signals travel from the olfactory bulb to the piriform cortex, which projects to the ventral insula and orbitofrontal cortex [19]. The insular and orbital regions involved in this process are also strongly connected to the amygdala and anterior cingulate cortex (ACC) [20], [21]. When food enters the stomach, neural signals from the gastrointestinal tract travel via the vagus nerve to the brainstem and thalamus, which projects to the rest of the brain in particular the hypothalamus, amygdala and primary sensory cortices [22].

The integration of sensory and gastric signals in the brain is difficult to study, especially in humans, because of their complexity and methodological challenges. Sensory perception is relatively well studied [23]–[25], but only a few well-controlled neuroimaging studies have examined stomach distention per se [26], [27]. In the latter studies brain activation was observed in the insula, amygdala, posterior insula, left inferior frontal gyrus and ACC [26], [27]. To our knowledge, the different contributions of oral stimulation and gastric distention by food on brain activation have not been investigated. Moreover, to date the process of satiation has not been examined in the brain in real time.

In addition to neural signals, hormonal signals are important for meal termination. Peptides secreted from the gastrointestinal tract interact with gastric as well as sensory signals during food intake [28]–[30] and provide information to the brain which leads to inhibition or stimulation of food intake [31], [32]. Gut peptides like ghrelin and cholecystokinin-8 (CCK-8) act on vagal afferents, the brainstem and other brain areas [33]–[35], in particular the hypothalamus. However, hormonal responses to food administration have rarely been linked to brain responses in humans (e.g., [36]).

Therefore, the aim of this study was to image the brain areas involved in the process of consumption, during and after food administration. The primary objective was to investigate the acute effects of gastric distention with a nutritious load on brain activity, and to assess the influence of oral stimulation on brain activity after food administration. The secondary objective was to determine to which extent changes in appetite hormone concentrations and subjective appetite-related ratings correlate with brain activation. First, we hypothesized that gastric distention (water or chocolate milk) will evoke activation in the midbrain, hypothalamus, insula, and ACC and that distention of the stomach by infusion of nutrients will activate the striatum in comparison with the non-caloric load. Second, we hypothesized that oral administration will activate reward areas, such as the striatum and amygdala more than infusion of chocolate milk. Finally, we expected a correlation between hormonal changes and hypothalamus activation.

## Materials and Methods

### Ethics Statement

The experimental procedures were explained in detail to the subjects. Prior to participation written informed consent was obtained from all subjects. Ethical approval was obtained from the Medical Ethical Committee of the University Medical Center Utrecht in accordance with the Declaration of Helsinki (ABR #35991). This trial was registered at clinicaltrials.gov as NCT01644539.

### Subjects

The study was performed at the MRI facility of the University Medical Center Utrecht, The Netherlands. Subjects were recruited by flyers posted at the University Medical Center Utrecht and Utrecht University campus in the spring of 2011. Sixteen healthy-normal weight volunteers participated in the study, of which fourteen were included in the final analyses (two subjects were withdrawn due to discomfort associated with the naso-gastric tube). Subjects were right-handed males, with an average age of 24.6±3.8 yr, and an average body mass index of 22.3±1.6 kg/m^2^. Exclusion criteria included: disliking chocolate milk, smoking, slimming or following a medically prescribed diet, restrained eating [37], [38], having an eating disorder, having a history of or current alcohol consumption >28 units per week, or any diseases (including neurological and psychiatric diseases, and taste and smell disorders), use of medication, and the presence of any metal objects within the body, or other contraindications for MRI. Subjects were informed about their eligibility and the procedure and risks were explained. When subjects met the inclusion criteria they were invited for a training session. After the training sessions, subjects could decide to withdraw or to proceed with the functional magnetic resonance imaging (fMRI) sessions. Subjects received adequate financial compensation for participation. Treatment sessions started August 2011 and data collection ended December 2011. On the basis of other fMRI studies [26], [27], [39], [40] power analysis with the G*Power program (version 3.1; Heinrich-Heine-Universität, Düsseldorf, Germany) showed that with an effect size = 0.5, α = 0.05 and β = 0.10 (power = 1 - β = 0.90), >11 subjects were needed.

### Design

**Figure 1:**
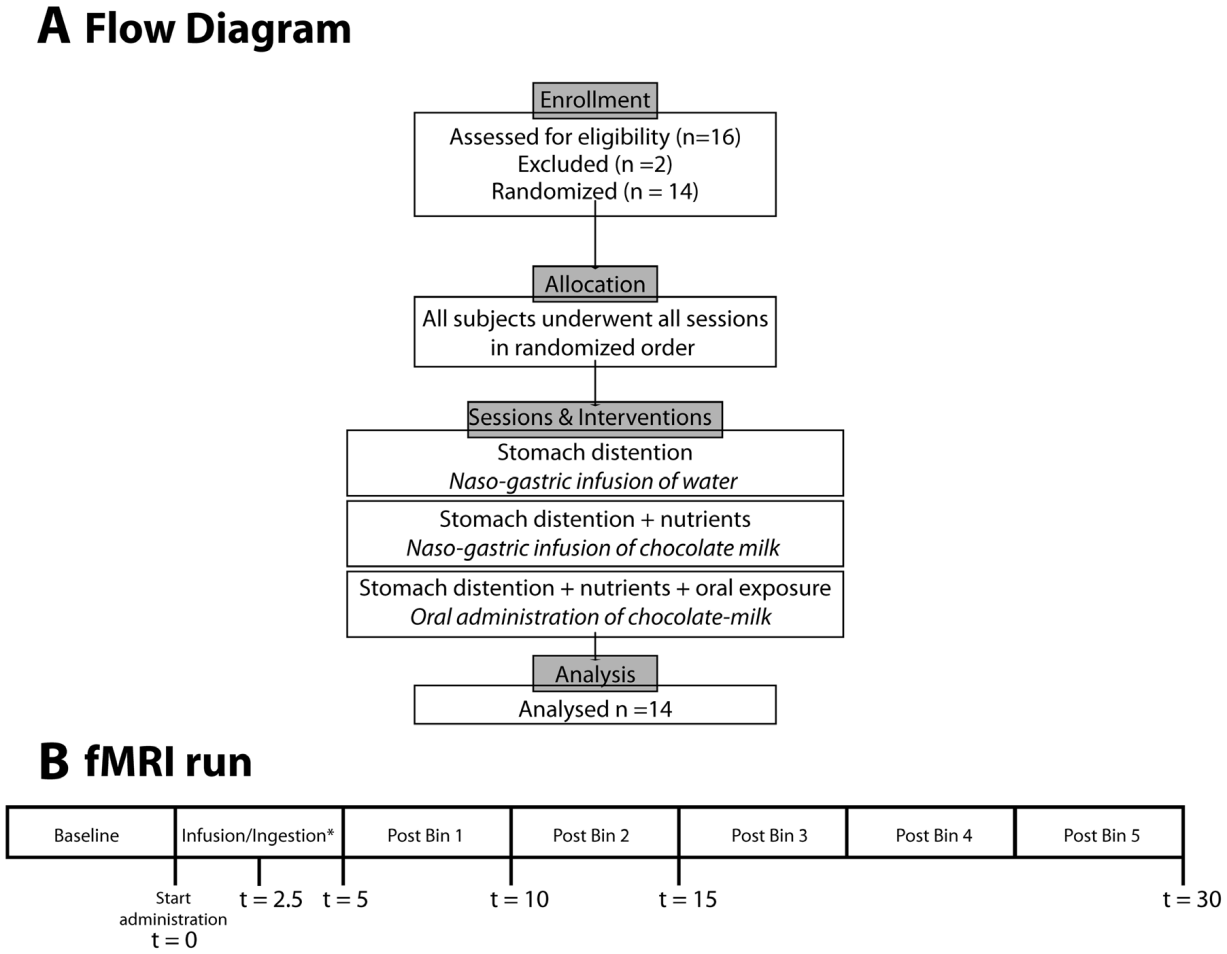
Experimental design. A: Flow diagram. B: Timeline of events during one fMRI run (total duration 35 min). Every block represents one 4.5-min time bin. At all illustrated time points (t = min) blood was drawn and fullness, desire to eat and anxiety were rated.*During this time bin chocolate milk was infused or ingested, or water was infused.

The study had a randomized, single blind, crossover design with three experimental conditions: naso-gastric water infusion, naso-gastric chocolate milk infusion, and oral chocolate milk administration (Figure 1A). Subjects were randomly allocated to a selected treatment order, based on enrollment in the study. Six different orders could be assigned. The three treatment sessions were scheduled on three separate days, at least 1 week apart in a time period of 2 months. Subjects were not aware of the order they were assigned to, and were unaware of the content of the load during the gastric infusions. The three sessions were conducted at least one week apart in a time period of two months per subject.

### Stimuli

Two different stimuli were used. During the training session, and two of the fMRI sessions (oral and gastric caloric) a caloric load consisting of chocolate milk (Chocomel, FrieslandCampina, Ede, the Netherlands, per 100 mL: energy content of 354 kJ, 3.5 g proteins, 12 g mono and disaccharides, 2.5 fat g, 0.5 g fibers) was used. Water was used for the gastric non-caloric session. To adjust for viscosity differences, 1% guar gum (E412) was added to the water. In a pilot study we established that in our setup, i.e., a nasogastric tube with a peristaltic pump, at 1% guar gum the rate and timing of the stimulus delivery was equal to that of the chocolate milk.

### Stimulus delivery

The loads were administered with a computer-controlled peristaltic pump (323DU, Watson-Marlow Ltd, Falmouth, Cornwall, UK), so as to simulate a normal drinking pattern (sips rather than continuous infusion). The pump was used with a silicon tube (inner diameter 4.8 mm, outer diameter 8 mm). In the oral session, one end of tube was placed between the lips of the subject. In the gastric and control sessions this tube was connected to the naso-gastic tube (Nutricia Flocare, Nutricia Medical Devices BV, Schiphol Airport, The Netherlands, length 110 cm with an inner diameter of Ch8 = 2.67 mm). The pump was programmed to deliver 100 mL/min with a sip size of 12 mL (delivered in 3 s) followed by a 4 s delay (for swallowing). Sip and swallow onset were cued on a screen during every session. Subjects were acquainted with these procedures in a training session and could stop the pump any time by pressing a button. First, 250 mL was ingested in 2.5 min followed by a pause of 30 s. Subsequently, another 250 mL was ingested after which subjects again gave their ratings. All instructions were displayed on a screen through a computer interface, run by the computer program PRESENTATION (Neurobehavioral Systems Inc, www.neurobs.com).

### Procedure

#### Training session

During the training session subjects were asked to come in fasted for at least two hours. A nurse inserted a naso-gastric tube. After insertion of the tube, subjects rested to allow the water used to facilitate insertion of the tube to leave the stomach and to become comfortable with the tube. During tube insertion the nurse and the subject evaluated how well the naso-gastric tube was tolerated. To simulate the position in the MR scanner, subjects were asked to lie down on an exam table. A tube was placed between the teeth of the subject (like a straw) after which an oral load of 500 mL was ingested. Ingestion was driven by a computer-controlled pump (as described in the stimulus delivery section). During this session subjects became familiar with the drinking procedure (drinking supine in a controlled manner).

#### fMRI sessions

After an overnight fast, subjects came into the lab in the morning between 8 am and 11 am. All subjects were asked to consume the same ready meal the evening before all three sessions, and were not allowed to eat anything after 10pm (subjects were asked to bring in the packaging of their ready meal every session). Subjects underwent three treatments in random order: Stomach distention, which consisted of naso-gastric infusion of 500 mL/0 kJ water (+ guar gum); Stomach distention with caloric content, which consisted of naso-gastric infusion of 500 mL/1770 kJ chocolate milk; Stomach distention with caloric content and oral exposure, which consisted of oral administration of 500 mL/1770 kJ chocolate-milk. Upon arrival, an appetite questionnaire was filled in (hunger, fullness, thirst, desire to eat, prospective consumption, desire to eat something sweet or savory, nausea, anxiety) on a 100-mm Visual Analog Scale (VAS) anchored with ‘not at all’ on the left side and ‘very much’ on the right side. In every session a qualified nurse placed a naso-gastric tube and an intravenous (i.v.) canula, after which the appetite questionnaire was filled in again. Subsequently, the subject was placed in the scanner and the baseline blood draw was obtained (t = 0). A towel was placed underneath the subject's left side, such that the stomach position resembled that in an upright position in order to approximate normal gastric filling. First, a 5-min anatomical scan was obtained, after which the 35-min fMRI scan started (Figure 1B). The first five minutes of the fMRI scan constituted the baseline measurement after which the administration of the stimuli started; either the oral load through a tube held between the lips, or an intra-gastric load through the naso-gastric tube. The start of ingestion of the load was defined as t = 0. At t = 2.5, 5, 10, 15 and 30 min further blood draws were obtained. At these same time points subjects rated their anxiety, desire to eat and fullness on a VAS by use of a button box. After the fMRI scan the subject was taken out of the scanner and the naso-gastric tube and i.v. canula were removed and another appetite questionnaire was filled in.

### Blood sampling and analysis

Blood samples were collected right before treatment onset (t = 0), and at t = 2.5, 5, 10, 15 and 30 min. After each blood draw, 2 mL of physiological salt solution (NaCl 0.9%) was injected into the canula to prevent it from clotting; before each blood draw an extra tube was drawn to remove the physiological salt solution.

Plasma for ghrelin and CCK-8 was collected in EDTA tubes which also contained a proprietary cocktail of protease, esterase and DPP-IV Inhibitors and were kept on ice. Plasma was obtained by centrifugation (1000×g/3000 rpm for 10 min at 4°C). Plasma was stored in aliquots at −30°C before analysis. Glucose concentrations were analyzed with the hexokinase method (Glucose HK 125 kit, Abbott). Active and total ghrelin were measured using human ELISA kits (Millipore RIA GHRT-88HK, Billerica, MA, USA). Plasma CCK-8 concentrations were measured using a commercial RIA kit (Eurodiagnostica RIA RB302, Malmö, Sweden).

The lowest detection limit for active ghrelin was 3.9 pg/mL, the intra-assay CV was 10% at mean concentrations of 1000 pg/mL and 4.4% at 3000 pg/mL and the inter-assay CV was 14.7% at 1000 pg/mL and 16.7% at 3000 pg/mL. The lowest concentration of total ghrelin that could be detected was 82 pg/mL. The intra-assay CV was 9.5% at 235.76 pg/mL and 6.7% at 138.56 pg/mL. The inter-assay CV at these same concentrations was 13.7% and 9.6% respectively.

Plasma CCK-8 concentrations were measured using a commercial RIA kit (Eurodiagnostica RIA RB302, Malmö, Sweden). The lowest detection limit of this RIA assay was 0.1 pmol/L. The inter-assay CV was 13.7% at mean concentrations of 4.2 pmol/L, and 2.0% at 20.6 pmol/L. The intra-assay CV for the same mean concentrations was 5.5% and 2.0%.

All analyses within one subject were done in one run. For all analyses concentrations below the detection limit were set at the lower detection limit. This occurred in 6 out of the 252 samples and only at t = 0 and 2.5 min. When concentrations were above the highest concentration of the calibration curve, measurements were added as missing value in the statistical analyses. This occurred for two subjects in the intra-gastric caloric condition for CCK-8 and insulin at t = 5, 10 and 15 min.

### fMRI data acquisition

MRI scans were performed on a 3-Tesla Philips Achieva at the University Medical Center Utrecht. First a T_1_-weighted anatomical scan was acquired (TR/TE = 61/8.4 ms, flip angle = 30°, FOV = 288×175 mm, 175 axial slices, voxel size = 1×1×1 mm). Next, a functional MRI scan was made (2D gradient echo EPI sequence, TR/TE = 1400/23 ms, flip angle = 70°, FOV = 208×120×256 mm, 43 interleaved axial slices, voxel size = 4×4×4 mm). The duration of each functional scan was 35 min, during which 1490 volumes were obtained.

### fMRI data processing and analysis

The neuroimaging data were preprocessed and analyzed using the SPM8 software (Wellcome Department of Imaging Neuroscience, London, UK, http://www.fil.ion.ucl.ac.uk/spm/software/spm8/) run with MATLAB 7.5 (The Mathworks Inc, Natick, MA)) using standard procedures [41]. First, all functional volumes of every subject were aligned with the first volume of the first run. Second, the images were normalized (retaining 4×4×4 mm voxels) to Montreal Neurological Institute space (MNI space) [42], and spatially smoothed with a Gaussian kernel of 8 mm full width at half maximum.

#### Subject level analyses

Subject level analyses were performed by splitting every functional run into seven 4.5-min time bins (based on seven consecutive 5-min time bins with 30 s of rating excluded per time bin): One pre-treatment bin (baseline bin T0), one treatment bin (T1) and five post treatment bins (T2–6). A regressor was created to separate instructions and ratings from the other bins; this was neglected in subsequent analyses. For each subject and condition the blood oxygen level-dependent (BOLD) signal averages for the treatment and post-treatment bins were compared with the baseline bin, using regression analysis resulting in six contrast images per scan session [43], [44].

#### Group level analyses

All 18 contrasts images from all subjects from the subject level analyses were entered into a 6 time (T1–T6)×3 conditions repeated measures ANOVA in SPM8 [43], [44]. This model was used to test for the main effect of gastric infusion, and differences in brain response between the conditions. Unpredicted peaks were considered significant at *P*<0.05 (FWE-corrected for multiple comparisons across the whole brain). Regions of interest (ROIs) were gastric distention and reward areas found in previous studies which include the amygdala, insula, inferior frontal gyrus, anterior cingulate cortex, hypothalamus and striatum [26], [27], [45]. These ROI masks were made using the WFU Pickatlas tool [46], and were considered significant at *P*<0.05 (FWE-corrected for multiple comparisons across the whole brain).

In subsequent analyses, to determine if the neural responses (change from baseline) was correlated with changes in hormone levels and VAS ratings (from baseline) we added the change in hormone concentration and VAS ratings at four time points (5, 10, 15 and 30 min) as covariates to an ANOVA model with the four corresponding time bins. Correlations with a P<0.001, uncorrected for multiple comparison, k>11, were considered significant. For all significant clusters mean parameter estimates for each cluster were obtained with the use of the MarsBaR toolbox (http://marsbar.sourceforge.net/). Subsequently, the correlation coefficient (r) was calculated with the use of SPSS 19.

## Results

### Brain activation

**Figure 2:**
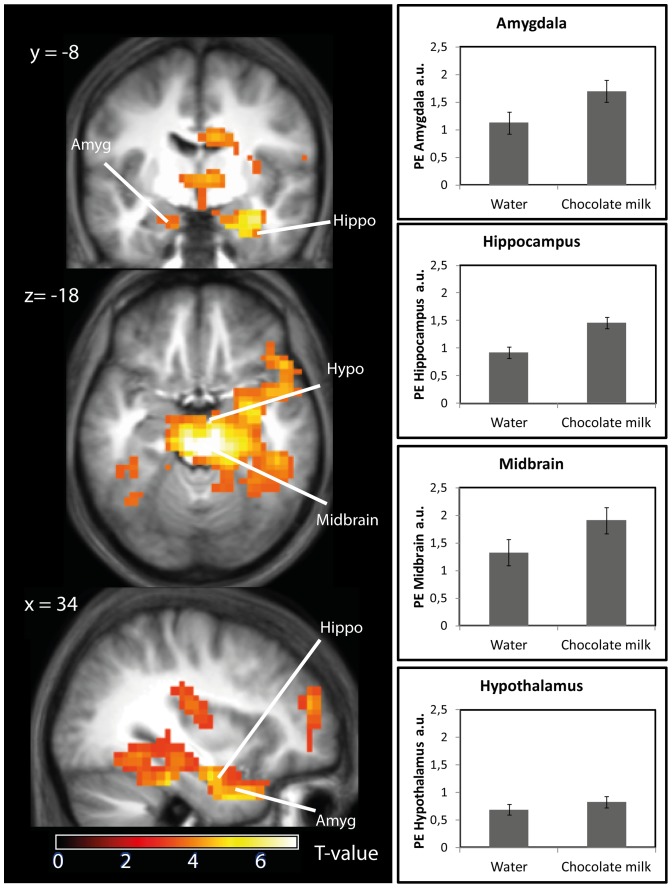
Effect of gastric infusion of water and chocolate milk on brain activity compared to baseline. Left panel: T-map of the increased response to chocolate milk and water infusion versus baseline overlaid onto the mean anatomical scan, thresholded at P<0.05, FWE-corrected for multiple comparison. Right panel: Mean parameter estimates (a.u. ± SEM) within significant clusters for water and chocolate milk infusion.

The main effect of gastric distention was increased brain activity during chocolate milk and water infusion in the hypothalamus (peak voxel MNI (−12, 2, −8), z = 4.66, amygdala (peak voxel MNI (26, −4, −26), z = 5.32,), hippocampus (peak voxel MNI (34, −8, −22), z = 5.76, and midbrain (peak voxel MNI (6, −28, −18), z >7) (Table 1 and Figure 2) compared to baseline.

**Table 1:** Effect of gastric infusion on brain activation in healthy normal-weight young men.

Region^1^	Peak voxel coordinates^2^			z-score
	x	y	z	
Midbrain	6	−28	−18	>7
	14	−15	−2	6.05
Hippocampus	34	−8	−22	5.76
	25	−24	−14	4.56
	18	−24	−10	4.54
Amygdala ROI	26	−4	−26	5.32
	−26	0	−26	3.83
	−22	−8	−22	3.32
Hypothalamus ROI	12	2	−8	4.66
	2	−8	2	4.09

1 Values are clusters of mean brain activation, n = 14. Reported clusters were thresholded at P<0.05 (FWE-corrected for multiple comparisons).

2 Voxel coordinates are in MNI space [42].

There was no difference in brain activation between chocolate milk and water infusion. We have no valid data of the brain activity during ingestion of chocolate milk, due to movement artefacts associated with swallowing.

**Figure 3:**
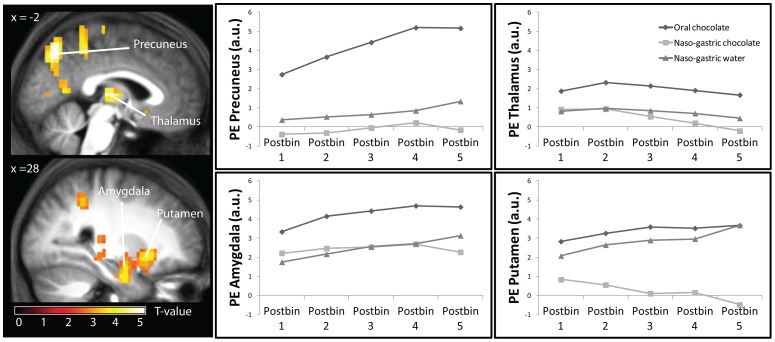
Changes in brain activity after treatment for the three conditions. Left panel: T-map of the increased response to oral chocolate milk stimulation after administration versus baseline overlaid onto the mean anatomical scan, thresholded at P<0.05 (FWE-corrected for multiple comparison). Right panel: Mean parameter estimates (a.u. ± SEM) over time from selected significant clusters. Area under the curve was greater for the oral condition in all brain areas, and for the control condition in the putamen (all P<0.05).

In the 20 minutes following infusion or ingestion activity in the putamen (peak voxel MNI (22, 12, −10), z = 5.07) was greater after water- than after chocolate milk infusion. Additionally, brain activity in the amygdala (peak voxel MNI (34, 0, −26), z = 4.67, FWE-corrected), thalamus (peak voxel MNI)-2, −16, 6), z = 5.24, FWE-corrected), left precuneus (peak voxel MNI (−2, −68, 50), z = 4.98, FWE-corrected) and putamen (peak voxel MNI (26, 16, −6), z = 4.57, FWE-corrected) increased more after ingestion (oral condition) than after gastric chocolate milk infusion compared to baseline, whereas the ACC (peak voxel MNI (6, 28, 15), z = 5.15, FWE-corrected) and hippocampus (peak voxel MNI (34, −8, −26), z = 5.65, FWE-corrected) evoked greater activation in the gastric chocolate milk condition (see Table 2 and Figure 3).

**Table 2:** Effect of treatment with chocolate milk on brain activation in healthy normal- weight young men.

Condition	Region^1^	Peak voxel coordinates^2^			z-score
		x	y	z	
**Oral > gastric**	Thalamus	−2	−16	6	5.24
		18	−24	−10	4.74
	Precuneus	−2	−68	50	4.98
		10	−54	52	4.65
		10	−72	46	4.12
	Amygdala ROI	34	0	−26	4.67
		26	−4	−26	4.47
		30	−4	−14	4.47
	Putamen ROI	26	16	−6	4.57
		30	0	−10	3.74
**Oral < gastric**	HippocampusROI	34	−8	−26	5.65
		−30	−32	−2	4.61
	ACC ROI	6	28	14	5.15

Oral > gastric shows areas with increased activation in the oral condition, oral < gastric shows areas with increased activation during the gastric chocolate milk condition.

1 Values are clusters of mean brain activation, n = 14. Reported clusters were thresholded at P<0.05 (FWE-corrected for multiple comparisons).

2 Voxel coordinates are in MNI space [42].

### Correlation between brain activation and changes in subjective ratings

**Figure 4:**
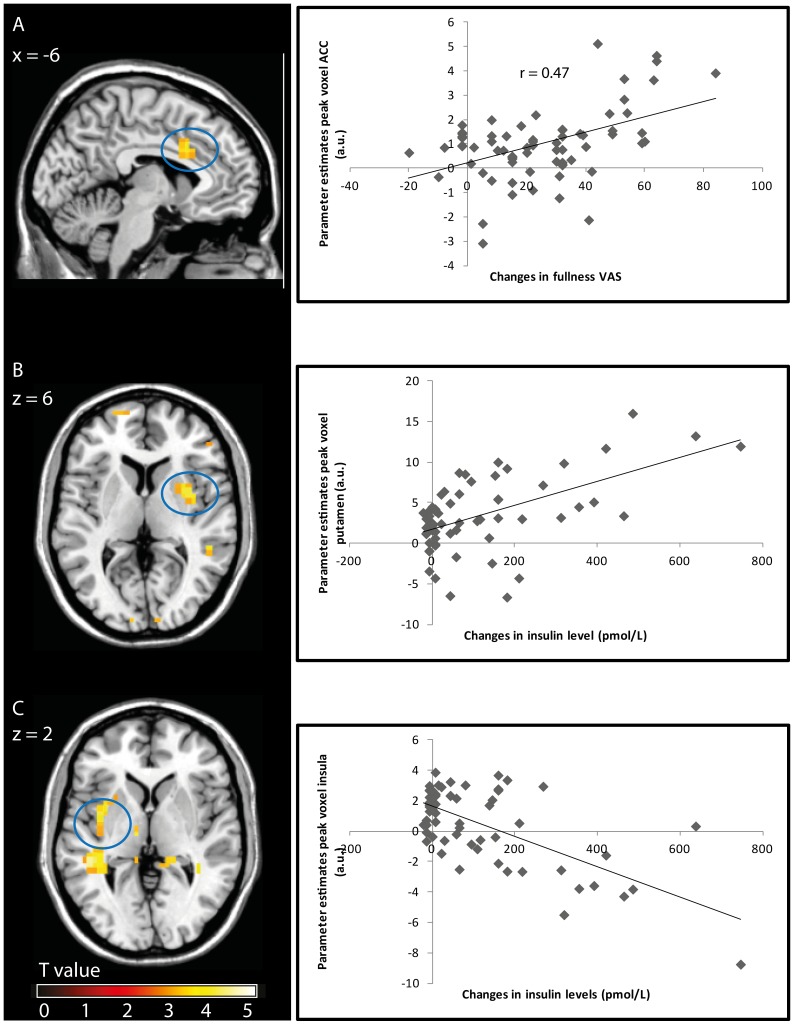
Correlation between fullness and insulin changes (from baseline) and changes in brain activity in corresponding time bins during the gastric condition (n = 14, 5 time bins per subject, T-maps are thresholded at P<0.001, uncorrected for multiple comparisons). Left pane: T-map of selected significant correlations overlaid onto the mean anatomical scan. A: Correlation T-map and scatter plot showing the parameter estimates of the ACC peak voxel at MNI (−6, −16, 30) against fullness changes. B: Correlation T-map and scatter plot showing the parameter estimates of the putamen peak voxel (34, 0, 6) against insulin changes. C: Correlation T-map and scatter plot of the parameter estimates of the insula peak voxel at MNI (−38, 0, 2) plotted against insulin changes.

Changes in fullness ratings (Table S1) during the naso-gastric infusion of chocolate milk were positively correlated with ACC activation (peak voxel MNI (−6, −16, 30), z = 3.72, FWE-corrected P = 0.022, r = 0.47) (Figure 4). There were no correlations with changes in the desire to eat. Additionally, in the conditions with water infusion and oral administration of chocolate milk there were no significant correlations between changes in brain activity and any of the subjective ratings.

### Correlation between brain activation and hormone responses

Hormone responses are tabulated in Table S2. In the session with naso-gastric infusion of chocolate milk, insulin changes correlated positively with putamen activation (peak voxel MNI (34, 0, 6), z = 3.81, FWE-corrected P = 0.011, r = 0.56), and negatively with middle and posterior insula activation (peak voxel MNI (−38, 0, 2), z = 3.81, FWE-corrected P = 0.047, r = −0.67) (Figure 4). Brain activation in the other conditions did not correlate with insulin changes. CCK-8, glucose, total and active ghrelin responses did not correlate with brain activation in any of the conditions.

## Discussion

We investigated the effect of matched non-continuous gastric infusion and ingestion on brain activation. The direct effect of infusion was independent of the nutrient content of the load. In addition, we observed differential brain responses after administering chocolate milk orally and gastrically.

We found that stomach filling evoked increased activity in the midbrain, hypothalamus, amygdala and hippocampus. There was no significant difference between the water and chocolate milk conditions. Thus, this response is driven by stomach filling rather than the load's macronutrient content. Normally when one is eating, cephalic neural signals travel from the brainstem to the thalamus, which projects to the rest of the brain, in particular the hypothalamus, amygdala and primary sensory cortices [22], [47], [48]. Additionally, from the midbrain and hypothalamus, areas which are involved in maintaining homeostasis and the regulation of energy balance [48], neural signals are directed, among other areas, to the amygdala and hippocampus. These latter regions play an important role in reward [49] and emotion processing [50] in relation to feeding behavior [51] and in signaling satiety [45], [52]–[54]. We observed that these areas responded to gastric infusion. Thus stomach distention alone, regardless of the nutrient content of the load and in the absence of oral exposure, is sufficient to increase brain activity in these reward and eating behavior-related areas. This is partly in line with the results of Wang et al. [26], who observed amygdala activation during repeated stomach distention with a balloon filled with up to 500 mL water. They also found that subjective fullness ratings correlated with amygdala activation. In our single-load design with non-continuous gastric infusion, there was no correlation between experienced fullness and amygdala activation. Additionally, animal work has shown that gastric distention with a gastric balloon can increase brain activity in homeostatic areas [55]. Moreover, BOLD signal changes in this study were highly correlated with increases in blood pressure [55]. This raises the possibility that gastric distention-related BOLD signal changes may in part be attributable to concomitant increases in blood pressure. Therefore, future studies should strive to incorporate blood pressure measurements. The question remains whether activity in these reward- and eating-related areas would be similar during normal ingestion (or during gastric filling combined with orosensory stimulation). Unfortunately, brain responses during oral food administration could not be assessed, due to movement-related artifacts caused by swallowing.

Surprisingly, after treatment both water infusion and chocolate milk ingestion caused greater putamen activation than chocolate milk infusion did. The putamen is involved in the expectation of reward [56], [57]. Gastric administration of chocolate milk and subsequent detection of calories in the gastrointestinal tract may constitute an unexpected metabolic reward, due to the lack of preceding oral sensory stimulation and swallowing. The enhanced putamen response after water infusion shows that, also in the absence of calories, sudden stomach filling can elicit reward-related activation. This suggests that gastric signaling can be dominated by vagal reflexes that do not rely on nutrient detection, in accord with the study of Wang et al. [26] in which the amygdala responded to gastric distention with a balloon.

After treatment, oral administration of chocolate milk evoked greater activation in the thalamus, precuneus, and amygdala than naso-gastric infusion of chocolate milk. These areas are involved in sensory perception [58] including taste processing [17]. The thalamus is a sensory relay area involved in the preparatory (food seeking) aspects of eating behavior [59] which receives gustatory and gastrointestinal inputs and projects to the cortical gustatory areas [60], [61]. The amygdala is involved in processing aversive and rewarding stimuli [49], [62]. It is sensitive to the salience of food cues, which is influenced by subject's internal state [49], [63]. In the previously mentioned study in which the stomach was distended with a balloon in a block design, these same areas were activated (amygdala, thalamus and left precuneus), along with increasing fullness ratings [26]. Here, we used fluid stimuli to distend the stomach, to mimic normal water or chocolate milk intake. Therefore, differences between conditions may reflect differences in gastric emptying rate. Unfortunately, our fMRI setup precluded measurement of gastric emptying. However, other studies have shown that liquid loads infused into the stomach can evoke faster gastric emptying compared to oral consumption [14], [15]. Also, the presence of fat slows down gastric emptying [14]. Therefore, even though the rate of delivery to the stomach was the same in all three conditions, it is likely that the gastric emptying rate was higher for the control stimulus due to a lack of macronutrients [14], [15]. This can also explain the difference in VAS and hormone release [64]. Hence, differences in gastric emptying rate and accompanying differences in the degree of gastric distention may explain neural differences between the water and the chocolate milk conditions. This could be mediated by differences in hormone responses, although we found little correlation, but also e.g by differences in blood pressure linked to gastric distension, which may affect the brain activity [55], [65]. Accordingly, the increased activation of the thalamus, precuneus, and amygdala by an oral nutritious load may be attributed to differences in sensory stimulation, associated heightened attention, and presumably slower gastric emptying, which is also reflected in greater increased fullness ratings.

In the gastric condition, we found a positive correlation between the degree of postprandial ACC activation and changes in fullness ratings. ACC activation has been observed in several studies in which hunger state was altered [45], [66], [67]. Recently, we reported a negative correlation between taste activation of the anterior part of the ACC and subsequent ad libitum intake [67]. This, along with other studies [45], [68], suggests that ACC activation reflects the degree of satiety. Here, we extend this by showing that increased postprandial middle ACC activity is associated with greater changes in fullness in the absence of oral stimulation. The absence of this correlation in the oral condition may be due to subjects attending to the act of drinking and the associated sensory stimulation rather than to their gastric sensations. This effect may have been enhanced because subjects drank in a supine position and in a fixed rhythm, rather than entirely self-paced (although they could pause at will during ingestion).

We found that the insulin response in the gastric chocolate milk condition correlated negatively with insula activation. It has been shown that insula activity [54], [69] and activation in response to food cues [66], [68] are greater when subjects are hungry. When satiated, plasma insulin concentrations are negatively correlated with left insular activation [69]. Also, in a study where subjects consumed 75 g glucose, insulin changes correlated negatively with insula and ventral striatum activation during looking at food pictures [39]. Our finding that greater postprandial insulin excursions were associated with lowered insula activation concurs with these previous findings and may provide an explanation for reduced food cue-induced insula activation in the form of lowered baseline activity.

Putamen activation was positively correlated with insulin changes. It has been suggested that putamen activity reflects the motivation to eat [70]. In line with this, increased putamen activity has been observed after a 36-h fast [69]. Thus, we demonstrated that the insulin response, which is indicative of the amount of carbohydrate being absorbed, i.e. the degree of nutrient repletion, is proportional to changes in activity of this limbic area. Similar correlations were not found in the oral condition. This may be due to the smaller magnitude of insulin responses in the oral condition.

In conclusion, we have demonstrated that key areas involved in the regulation of food intake are activated by gastric filling, independent of nutrient content. Compared to gastric infusion, oral food administration evoked greater activation in several brain areas involved in gustatory and reward processing, and was associated with greater fullness and less desire to eat. There were few correlations between blood parameter responses and brain activation, and these were only found in the gastric chocolate milk condition. This underscores the great complexity of gut-hormone-brain interactions in normal food ingestion, which makes observing correlations between single hormone responses and brain activation unlikely. Thus, we have provided neural evidence for the importance of oral sensory stimulation for satiation and optimal digestion. Future research should further elucidate the complex interplay between oral sensory stimulation, gastric filling, hormone responses and brain responses.

## Supporting Information

Table S1 **Changes in subjective ratings during the three sessions.**
(PDF)

Table S2 **Changes in blood parameters during the three sessions.**
(PDF)

Checklist S1 **CONSORT checklist.**
(PDF)
