# Supplementary material for: The Sum of Its Parts—Effects of Gastric Distention, Nutrient Content and Sensory Stimulation on Brain Activation
Source: PLoS One. 2014 Mar 10;9(3):e90872. doi: 10.1371/journal.pone.0090872 (PMC3948722; doi:10.1371/journal.pone.0090872)
Supplement: Table S1 — Changes in subjective ratings during the three sessions. (PDF) [file pone.0090872.s001.pdf]

Supporting information

Table S1. Changes in subjective ratings during the three sessions[1].

Time point	Fullness (mm)			Desire to eat (mm)			Anxiety (mm)		
	Oral - Cal	*Gastric-Cal*	*Gastric-NonCal*	*Oral - Cal*	*Gastric - Cal*	*Gastric - NonCal*	*Oral - Cal*	*Gastric-Cal*	*Gastric-NonCal*
Baseline	23.43	18.07	20.40	72.57	70.92	72.93	7.93	8.07	7.00
Δ 2.5	28.0*	14.79	11.60	-14.71*	-2.36	-6.93	5.64*	-0.93	0.86
Δ 5	51.57*	29.79	26.93	-44.00*	-12.36	-12.26	5.64*	-0.21	2.29
Δ 10	35.29*	29.07	26.27	-29.00*	-9.50	-6.93	-2.93	-3.79	-2.00
Δ 15	32.29	24.07	23.60	-30.43	-8.07	-3.90	-2.93	-5.21	-4.86
Δ 30	21.47	25.50	13.60*	-20.43	-11.64	-0.93	-3.64	-4.50	-3.43

[1] Shown are mean baseline measurement (n = 14) and changes from baseline at 2.5, 5, 10, 15 and 30 minutes. * and ** represent significant differences with other conditions at that time point.
