# Supplementary material for: The Sum of Its Parts—Effects of Gastric Distention, Nutrient Content and Sensory Stimulation on Brain Activation
Source: PLoS One. 2014 Mar 10;9(3):e90872. doi: 10.1371/journal.pone.0090872 (PMC3948722; doi:10.1371/journal.pone.0090872)
Supplement: Table S2 — Changes in blood parameters during the three sessions. (PDF) [file pone.0090872.s002.pdf]

Supporting information

Table S2. Changes in blood parameters during the three sessions. [1]

Time point	Glucose (mmol/L)			Insulin (pmol/L)			CCK-8 (pmol/L)		
	Oral - Cal	*Gastric-Cal*	*Gastric - NonCal*	*Oral - Cal*	*Gastric - Cal*	*Gastric - NonCal*	*Oral – Cal*	*Gastric-Cal*	*Gastric - NonCal*
Baseline	4.76	4.58	4.84	31.06	31.72	35.19	0.38	0.39	0.53
Δ 2.5	0.08	0.00	0.09	-4.49	-5.00	-5.72	0.027	0.39	0.05
Δ 5	0.05	0.04	0.06	-5.50	0.00	-6.23	0.05	0.83	0.09
Δ 10	0.22	0.45	0.06	7.24	52.86	-6.23	0.76	2.17*	0.13
Δ 15	0.60	0.98	0.04	51.92*	149.24**	-6.73	1.09	1.09	0.13
Δ 30	1.65	1.69	0.08	209.41*	348.15**	-8.76	0.85	1.17	0.53

Time point	Ghrelin total (pmol/L)			Ghrelin active (pmol/L)			
	Oral - Cal	*Gastric - Cal*	*Gastric - NonCal*	*Oral - Cal*	*Gastric - Cal*	*Gastric - NonCal*	
Baseline	3132	3061	3132	293	316	340	
Δ 2.5	-16.18	56.63	71.13	-39.63	64.27	22.98	
Δ 5	-22.92	102.48	136.19	-4.66	9.66	60.94	
Δ 10	5.39	182.71	111.58	13.65	88.58	89.91	
Δ 15	-45.5	157.43	188.10	-45.62	-106.56	109.22	
Δ 30	-267.32	334.40	70.11	-2.00	61.27	79.25	

[1] Shown are mean baseline measurement (n = 14) and changes from baseline at 2.5, 5, 10, 15 and 30 minutes. * and ** represent significant differences with other conditions at that time point. Conversion from metric to SI units was done as follows: insulin mIU/L × 7.241 = pmol/L, total ghrelin pg/ml × 0.37 = pmol/l and, active ghrelin pg/ml × 0.33 = pmol/.
